# Co-feeding transmission in Lyme disease pathogens

**DOI:** 10.1017/S0031182014001486

**Published:** 2014-10-08

**Authors:** MAARTEN J. VOORDOUW

**Affiliations:** Institute of Biology, Laboratory of Ecology and Evolution of Parasites, University of Neuchâtel, Emile Argand 11, 2000 Neuchâtel, Switzerland

**Keywords:** *Borrelia burgdorferi*, co-feeding transmission, epidemiology, saliva-assisted transmission, tick-borne pathogens

## Abstract

This review examines the phenomenon of co-feeding transmission in tick-borne pathogens. This mode of transmission is critical for the epidemiology of several tick-borne viruses but its importance for *Borrelia burgdorferi sensu lato*, the causative agents of Lyme borreliosis, is still controversial. The molecular mechanisms and ecological factors that facilitate co-feeding transmission are therefore examined with particular emphasis on *Borrelia* pathogens. Comparison of climate, tick ecology and experimental infection work suggests that co-feeding transmission is more important in European than North American systems of Lyme borreliosis, which potentially explains why this topic has gained more traction in the former continent than the latter. While new theory shows that co-feeding transmission makes a modest contribution to *Borrelia* fitness, recent experimental work has revealed new ecological contexts where natural selection might favour co-feeding transmission. In particular, co-feeding transmission might confer a fitness advantage in the Darwinian competition among strains in mixed infections. Future studies should investigate the ecological conditions that favour the evolution of this fascinating mode of transmission in tick-borne pathogens.

## INTRODUCTION

Co-feeding transmission is a mode of transmission that has been reported for a wide diversity of vector-borne pathogens (Jones *et al.* 1987; Randolph *et al.* 1996; Mead *et al.* 2000; Higgs *et al.* 2005). With respect to tick-borne pathogens, this mode of transmission was first discovered for tick-borne viruses such as Thogoto virus (Jones *et al.* 1987) and tick-borne encephalitis virus (TBEV) (Alekseev and Chunikhin, 1990; Labuda *et al.* 1993*a*, *b*) and was subsequently described in *Borrelia burgdorferi sensu lato* (*s. l.*), the complex of spirochaete bacteria that causes Lyme borreliosis (Gern and Rais, 1996; Randolph *et al.* 1996). While the importance of co-feeding transmission for TBEV epidemiology is now widely accepted (Randolph, 2011), the role of co-feeding transmission in the epidemiology of *B. burgdorferi s. l.* is more controversial (Randolph *et al.* 1996; Richter *et al.* 2002, 2003; Randolph and Gern, 2003). The controversy of whether co-feeding transmission is ecologically relevant to *Borrelia* pathogens has recently been invigorated with a number of theoretical and experimental studies. Theoretical work on the basic reproductive number of tick-borne pathogens suggests that co-feeding makes a modest contribution to *Borrelia* fitness but that spirochaetes can invade tick populations without this mode of transmission (Hartemink *et al.* 2008; Harrison *et al.* 2011; Harrison and Bennett, 2012. In contrast, the fieldwork suggests that co-feeding transmission may enhance *Borrelia* fitness in vertebrate hosts that are otherwise refractory to systemic infection by spirochaetes (Morán Cadenas *et al.* 2007; Kiffner *et al.* 2011; Kjelland *et al.* 2011). Experimental infection work has found evidence for genetic variation in co-feeding transmission among strains of *Borrelia* suggesting that this trait can evolve in response to natural selection (Tonetti and Gern, 2011). Thus co-feeding transmission could influence the Darwinian competition among strains for transmission success and by extension, the genetic community of *Borrelia* strains in the populations of the tick vector and the reservoir host (Pérez *et al.* 2011). In addition, co-feeding transmission may facilitate contact between *Borrelia* genospecies that are adapted to different vertebrate host species (Kurtenbach *et al.* 2001; Pichon *et al.* 2003; Herrmann *et al.* 2013). Thus co-feeding transmission may allow genetic exchange between *Borrelia* pathogens that are otherwise genetically isolated. In the present review, I discuss the ecological significance of co-feeding transmission and the underlying molecular mechanisms with particular emphasis on its importance to *Borrelia* pathogens.

## CO-FEEDING TRANSMISSION AND TICK-BORNE PATHOGENS

### Definition of co-feeding transmission of tick-borne pathogens

**Fig. 1.**
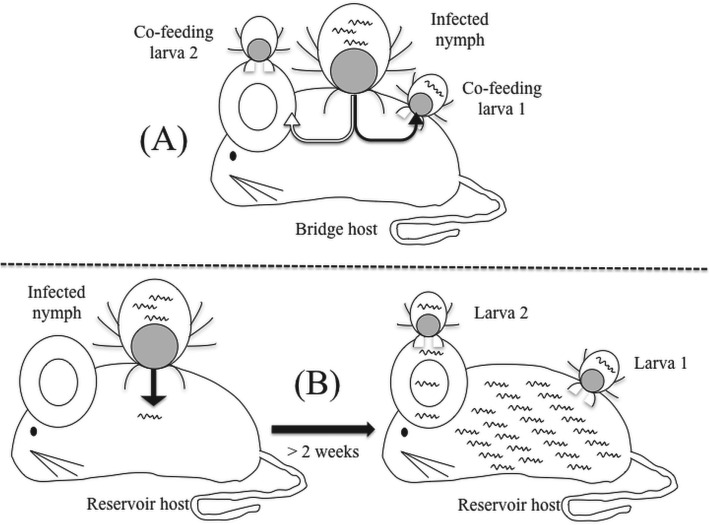
The diagram shows (A) co-feeding (nymph-to-larva) transmission and (B) systemic (host-to-larva) transmission of *Borrelia* spirochaetes in a rodent reservoir host. Co-feeding transmission can occur when ticks feed in close spatial and temporal proximity on the same host. Larva 2 does not acquire spirochaetes via co-feeding transmission because it is too far away from the infected nymph. Systemic transmission occurs once the spirochaetes have had enough time to disseminate to all the relevant tissues of the reservoir host, which usually takes about 2 weeks. Under systemic transmission, larvae can acquire spirochaetes by attaching anywhere on the infected mouse.

Co-feeding transmission is a mode of transmission of vector-borne pathogens that is distinct from systemic transmission (Fig. 1). Co-feeding transmission occurs when infected and uninfected vectors feed in spatiotemporal proximity to each other on the same reservoir host (Randolph *et al.* 1996; Randolph, 2011). This mode of transmission may be particularly significant for tick-borne pathogens because ticks, unlike other arthropod vectors, often attach to the host for several days to obtain a meal (Randolph, 1998; Nuttall, 1999). Co-feeding transmission often depends on an ephemeral, localized infection in the skin and is distinct from systemic transmission where the vector-borne pathogen disperses from the initial bite site and establishes a widespread (systemic) infection in the host organism (Fig. 1). In co-feeding transmission, the host acts as a transient bridge that brings infected and uninfected ticks together in the same time and place to facilitate pathogen exchange (Randolph, 2011). By contrast, in systemic transmission, the infected host acts as a reservoir from which vectors can acquire the pathogen for weeks or even months after the host became infected. In systemic transmission, there is often a latency period where the pathogen is replicating inside the host but the latter is not yet infectious to new vectors. By contrast, the latency period of co-feeding transmission is much shorter and is virtually instantaneous for some tick-borne viruses.

### Tick-borne pathogens capable of co-feeding transmission

Co-feeding transmission was first demonstrated in two tick-borne viruses: Thogoto virus (Jones *et al.* 1987) and TBEV (Alekseev and Chunikhin, 1990; Labuda *et al.* 1993*a*, *b*). These two arboviruses were both transmitted between co-feeding ticks without inducing detectable viral titres (viraemia) in the blood of their rodent hosts (Jones *et al.* 1987; Labuda *et al.* 1993*a*, *b*). Labuda *et al*. (1997) demonstrated that co-feeding transmission of TBEV can even occur on immunized rodents where sterilizing antibodies prevent the development of a viraemic infection. By knocking out systemic infection, this immunization experiment provided an elegant demonstration that co-feeding transmission is a distinct mode of pathogen transfer that can operate independently from systemic transmission (Labuda *et al.* 1997). Following its discovery in tick-borne viruses, co-feeding transmission was subsequently demonstrated in two groups of tick-borne bacteria: intracellular gram-negative bacteria belonging to the genus *Anaplasma* (formerly *Ehrlichia*) (Levin and Fish, 2000) and spirochaete bacteria belonging to the *B. burgdorferi s. l.* genospecies complex (Gern and Rais, 1996; Patrican, 1997; Sato and Nakao, 1997; Piesman and Happ, 2001; Richter *et al.* 2002; Hu *et al.* 2003). Interestingly, the *Anaplasma* genus exhibits species-specific differences in co-feeding transmission as the phenomenon was demonstrated in *Anaplasma phagocytophilum* (Levin and Fish, 2000) but not in the closely related *Anaplasma marginale* (Kocan and de la Fuente, 2003). In summary, co-feeding transmission has been demonstrated in a variety of tick-borne pathogens including viruses and bacteria.

### Co-feeding transmission in *B. burgdorferi s. l*

The *B. burgdorferi s. l.* genospecies complex contains a number of pathogens that cause Lyme borreliosis, the most common tick-borne disease in the Northern Hemisphere. Co-feeding transmission has been demonstrated for the three *B. burgdorferi s. l.* genospecies that are most commonly associated with human Lyme borreliosis: *B. burgdorferi sensu stricto* (*s. s.*) (Gern and Rais, 1996; Patrican, 1997; Piesman and Happ, 2001; Hu *et al.* 2003), *Borrelia afzelii* (Richter *et al.* 2002; Hu *et al.* 2003), and *Borrelia garinii* (Sato and Nakao, 1997; Hu *et al.* 2003), as well as *Borrelia valaisiana* (Hu *et al.* 2003). One reason for the controversial role of co-feeding transmission in Lyme disease is because systemic transmission of *Borrelia* spirochaetes from the reservoir host to the tick vector is highly efficient. For example, in the North American system of *B. burgdorferi s. s.* and the tick vector *Ixodes scapularis*, the systemic transmission rate from competent reservoir hosts such as the white-footed mouse, *Peromyscus leucopus*, can reach 90% (Donahue *et al.* 1987). By contrast, co-feeding transmission in this system was 20-fold lower (5%) and only occurred under very unrealistic tick infestation conditions (mice were infested with ~28 infected nymphs and 200 larvae) (Piesman and Happ, 2001). Co-feeding transmission of *B. burgdorferi s. s.* was higher in two other studies where the authors used either an unnatural gerbil reservoir host (18–88%) (Patrican, 1997) or European strains of *B. burgdorferi s. s.* in combination with *Ixodes ricinus* ticks (32·5–60·9%) (Gern and Rais, 1996). In the European system of *B. afzelii* and the tick vector *I. ricinus*, co-feeding transmission ranged from 1·6 to 55·3% under realistic tick infestation conditions (mice were infested with one infected nymph) (Richter *et al.* 2002). A study on field-collected *I. ricinus* ticks that were mostly infected with *B. afzelii* found that 95% (105/111) of all laboratory mice produced at least one co-infected tick (Hu *et al.* 2003) but unfortunately, the mouse-specific co-feeding transmission rates were not reported (Hu *et al.* 2003). A study on *B. garinii* and *Ixodes persulcatus* ticks found that the co-feeding transmission rates ranged from 6·0 to 29·0% (Sato and Nakao, 1997). While experimental differences in *Borrelia* genospecies, tick vector species and reservoir hosts make it difficult to generalize, co-feeding transmission appears to be more efficient in the European system of *B. afzelii* and *I. ricinus* than the North American system of *B. burgdorferi s. s*. and *I. scapularis*.

The viability of spirochaetes acquired via co-feeding transmission remains an open question. Many studies that measure co-feeding transmission use detection methods such as fluorescent antibody tests or PCR, which cannot establish whether the *B. burgdorferi s. l.* spirochaetes in the co-feeding ticks are actually alive (Gern and Rais, 1996; Patrican, 1997; Sato and Nakao, 1997; Richter *et al.* 2002). Evidence that co-feeding transmits viable *B. burgdorferi s. l.* comes from two studies that cultured live spirochaetes from co-feeding ticks (Piesman and Happ, 2001; Hu *et al.* 2003). However, in both of these studies, the spirochaetes were cultured in Barbour–Stoenner–Kelly (BSK) medium within 1 week of the co-feeding transmission event. In contrast, under natural conditions, *Borrelia* spirochaetes typically spend many months inside the nymphal tick before infecting a new vertebrate reservoir host. Thus the long-term survival prospects of co-feeding acquired spirochaetes in the tick vector remain unknown. Similarly, whether spirochaetes acquired via co-feeding transmission are infectious to vertebrate reservoir hosts also remains unknown.

## ECOLOGY OF CO-FEEDING TRANSMISSION

Larval and nymphal ticks maintain Lyme borreliosis in nature because these two immature tick stages feed on the same suite of reservoir hosts. Larvae (being the younger stage) are an order of magnitude more common than nymphs into which they develop following the larval blood meal. The generational transfer of *Borrelia* spirochaetes from a few infected nymphs to many uninfected larvae (via the host upon which they are feeding) is the critical life history event that defines the reproductive number (*R*_0_) and the epidemiology of Lyme disease (Randolph, 1998; Tsao, 2009). Transstadial maintenance of the infection, where infected, blood-engorged larvae maintain the infection during the moult and develop into the next generation of *Borrelia*-infected nymphs, is another essential feature of the spirochaete life cycle. Naive recipient larval ticks can acquire spirochaetes from feeding on an infected reservoir host (host-to-larva systemic transmission) or from feeding next to an infected donor nymph on a bridge host (nymph-to-larva co-feeding transmission). Nymph-to-nymph co-feeding transmission is possible (Patrican, 1997) but is much less common than nymph-to-larva co-feeding transmission. A field study on wild rodents in Slovakia found 12,032 attached larvae and 400 attached nymphs (Randolph *et al.* 1999). Thus in this particular rodent community, nymph-to-larva co-feeding transmission occurred 30 times more often than nymph-to-nymph transmission and the latter is therefore largely irrelevant to the fitness of tick-borne pathogens. Transovarial transmission has enormous potential to enhance spirochaete fitness because one infected female can produce many infected offsprings. However, two recent studies suggest that previous reports of transovarial transmission in *B. burgdorferi s. l.* were confounded by co-infection with *Borrelia miyamotoi*, a recently discovered species that belongs to the relapsing fever-group (Richter *et al.* 2012; Rollend *et al.* 2013). These new developments therefore suggest that transovarial transmission does not occur in *B. burgdorferi s. l.* (Richter *et al.* 2012; Rollend *et al.* 2013). The two key fitness components of *B. burgdorferi s. l.* pathogens are therefore the number of infected larvae produced via co-feeding transmission and the number of infected larvae produced via systemic transmission.

### Synchronous questing activity of immature ticks

Successful co-feeding transmission requires that larval and nymphal ticks feed at the same time and on the same host. Co-feeding transmission therefore has two necessary ecological conditions: synchrony of larval and nymphal host-searching (questing) activity and the co-occurrence of larvae and nymphs on the same host (Randolph *et al.* 1996, 1999). Differences in climate between North America and Europe produce contrasting tick activity patterns (phenologies) (Kurtenbach *et al.* 2006) with important consequences for co-feeding transmission. In North America, immature *I. scapularis* ticks exhibit asynchronous phenologies; peak nymphal and larval questing activities occur at different times of the year (early and late summer, respectively). By contrast, in Europe, immature *I. ricinus* ticks are active at the same time from spring to autumn (Craine *et al.* 1995; Kurtenbach *et al.* 2006; Burri *et al.* 2011). The potential for spirochaete co-feeding transmission is therefore probably much greater in Europe than in North America. A recent study in North America showed that climate-induced differences in the seasonal synchrony of tick questing activity can influence the community of circulating *Borrelia* strains (Gatewood *et al.* 2009). In the Northeast, a large temporal gap between peak nymphal and peak larval questing activity (i.e. high seasonal asynchrony) favours strains of *B. burgdorferi s. s.* that are long-lived inside the reservoir host (Gatewood *et al.* 2009). These long-lived strains are also more invasive in humans suggesting that interactions between climate, tick phenology and strain phenotype can have important consequences for the epidemiology of Lyme borreliosis.

Interestingly, climate change is predicted to have different consequences for co-feeding transmission on these two continents. In North America, climate change is expected to speed up the onset of larval activity patterns thereby increasing the scope for co-feeding transmission (Ogden *et al.* 2007). In Europe, by contrast, climate change is predicted to disrupt transmission cycles of tick-borne pathogens that are highly dependent on coincident feeding and co-feeding transmission (Randolph and Rogers, 2000; Randolph and Sumilo, 2007). For example, depending on the climate change scenario, TBEV will be largely eliminated from central Europe by 2050 (Randolph and Rogers, 2000; Randolph and Sumilo, 2007).

### Co-occurrence and aggregation of immature ticks on the same host

Co-occurrence of infected nymphs and susceptible larvae on the same host is another critical ecological condition for co-feeding transmission. Ticks are often highly aggregated on just a few hosts and follow the ‘20/80 Rule’ (Woolhouse *et al.* 1997) where 20% of the reservoir hosts feed about 80% of the immature ticks (Randolph *et al.* 1999; Perkins *et al.* 2003; Devevey and Brisson, 2012). In general, those host individuals that feed the greatest number of nymphs also tend to feed and infect the greatest number of larvae (Craine *et al.* 1995; Randolph *et al.* 1999; Brunner and Ostfeld, 2008). For example, a field study of wild rodents in Slovakia found that 26% of the most heavily infested individuals fed up to 75% of the nymphs and 86% of the larvae (Randolph *et al.* 1999). A field survey of yellow-necked mouse, *Apodemus flavicollis*, found that 20% of the mice (mostly adult males) fed 83% of the larvae and hosted 72% of the co-feeding events (Perkins *et al.* 2003). Similarly, a field survey on the wood mouse, *Apodemus sylvaticus*, found that 20% of the mice hosted all the nymphs and 72% of the larvae (Harrison *et al.* 2011). Calculation of the reproductive number (*R*_0_) for tick-borne pathogens such as TBEV suggests that these co-occurrence patterns of immature ticks on the same host increase pathogen fitness by a factor of three in comparison to the null hypothesis of independent larval and nymphal distributions (Randolph *et al.* 1999). Thus coincident feeding of immature ticks is critical for maintaining and amplifying co-feeding transmission.

There are a variety of reasons why ticks are aggregated on a subset of their hosts. Questing larvae are often highly aggregated in space because they hatch from a single egg batch and have limited dispersal (Steele and Randolph, 1985; Daniels and Fish, 1990). Male rodents tend to have higher tick burdens than female rodents because they are bigger and have larger home ranges (Randolph, 1975; Perkins *et al.* 2003). Another reason why male rodents are believed to be susceptible to high tick infestations is because their immune system is suppressed by testosterone (Hughes and Randolph, 2001). Estimates of tick burden and coincident aggregation are critical for parameterizing models that estimate the contributions of co-feeding and systemic transmission to the fitness of tick-borne pathogens (Harrison and Bennett, 2012).

### Mechanics of co-feeding transmission – time and distance

The efficiency of co-feeding transmission of *B. burgdorferi s. l.* depends on two important factors: the time between larval and nymphal fixation and the distance between the larval and nymphal attachment sites. To measure co-feeding transmission, workers typically place xenodiagnostic larvae on the host at the same time (Patrican, 1997; Sato and Nakao, 1997; Piesman and Happ, 2001) or a few days (2–5 days) after attachment of the *Borrelia*-infected nymphs (Gern and Rais, 1996; Richter *et al.* 2002; Hu *et al.* 2003). In the *B. afzelii*–*I. ricinus* system, co-feeding transmission increased from 0·0 to 55·3% as the duration of nymphal attachment before larval attachment increased from 0 to 3 days (Richter *et al.* 2002). Co-feeding transmission on a bridge host can take place even when the nymphs and larvae are not attached at the same time. In *B. burgdorferi s. s*. and the tick vector *I. ricinus*, co-feeding transmission from the site of infected nymphal attachment (the back of the mouse) occurred for 14 days, even after infected nymphs had detached, while systemic transmission from a distant site (the head) was not observed until 29 days following nymphal attachment (Gern and Rais, 1996). Thus systemic transmission is separated in time from co-feeding transmission.

The distance between co-feeding ticks is another factor that influences the efficiency of co-feeding transmission. Workers often place nymphs and larvae in capsules that are fixed to the skin of the bridge host to manipulate the distance at which ticks co-feed from each other (Gern and Rais, 1996; Sato and Nakao, 1997; Hu *et al.* 2003). In the *B. afzelii*–*I. ricinus* system, co-feeding transmission declines from 55·3 to 25·6 to 6·3% as the distance between nymphs and larvae increases from 0·0 to 1·0 to 2·0 cm (Richter *et al.* 2002). This spatial constraint would appear to reduce the importance of co-feeding transmission to spirochaete fitness. However, ticks do not randomly select feeding attachment sites and are often spatially clustered on the host. Most immature *Ixodes* ticks are found on the ears, head and neck of their rodent hosts (Randolph, 1975; Craine *et al.* 1995; Schmidt *et al.* 1999), presumably to avoid host grooming, which represents a significant source of tick mortality (Shaw *et al.* 2003; Keesing *et al.* 2009). A field survey of squirrels in England found that 95% of all immature *I. ricinus* ticks were found on the ears (Craine *et al.* 1995). Randolph suggested that ~45% of feeding ticks are within ~1 cm of each other on the rodent host, thereby greatly facilitating co-feeding transmission (Randolph, 2011). Spatial clustering of *I. ricinus* ticks was also observed on sheep in the northwest UK where 90% of the ticks were found on 20% of the sheep surface area (the part that was not covered by wool) (Ogden *et al.* 1998*a*). In these sheep populations, co-feeding is believed to be the predominant mode of spirochaete transmission (Ogden *et al.* 1997). A study on roe deer found that 54% of the total tick load was found on only 12% of the total surface area of the animals (Kiffner *et al.* 2011). Thus spatial clustering of *I. ricinus* larval and nymphal ticks is commonly observed in both rodents and ungulates.

In some tick species, co-occurrence on the same host and spatial clustering of ticks on the same host surfaces appear to be mediated by pheromones (Sonenshine, 2004). Spatial clustering may also facilitate cooperative feeding among ticks as demonstrated in several species of ixodid ticks (Wang *et al.* 1998; Rechav and Nuttall, 2000; Wang *et al.* 2001*b*). In *I. ricinus* for example, nymphs that co-fed with larvae had higher feeding success and greater engorgement weights than nymphs that did not co-feed with larvae (Ogden *et al.* 1998*b*). Cooperative feeding, by allowing vectors to pool their saliva, may enhance the immunomodulatory manipulation of the host organism. If the immunomodulatory constituents of tick saliva are costly, cooperative feeding could increase the cost-benefit ratio of resource extraction from the host relative to per capita investment in tick saliva production. Avoidance of host grooming behaviour, pheromone-induced aggregation and cooperative feeding are different mechanisms that enhance the spatial clustering of ticks on the same host. In turn, these spatial clustering mechanisms cause ticks to feed on the same patch of skin thereby enhancing co-feeding transmission of spirochaetes.

## MOLECULAR MECHANISMS OF CO-FEEDING TRANSMISSION

The molecular mechanisms that facilitate co-feeding transmission are better understood for TBEV than for *Borrelia* pathogens. Co-feeding transmission of TBEV appears to be mediated by migratory leucocytes. Langerhans cells, the dendritic cells that reside in the skin, appear to be recruited to the tick-feeding site where they acquire TBEV (Labuda *et al.* 1996). Infected Langerhans cells are believed to transmit the virus to T lymphocytes in the local lymph nodes (Nuttall, 1999; Nuttall and Labuda, 2003). The infected T lymphocytes are then recruited to the feeding sites of uninfected ticks thereby completing the co-feeding transmission cycle of TBEV (Nuttall, 1999; Nuttall and Labuda, 2003). Perhaps migratory leucocytes play a similar role in the co-feeding transmission of intracellular tick-borne bacteria such as *A. phagocytophilum* (Levin and Fish, 2000). *Borrelia*, being an extracellular bacterium, is therefore unlikely to use migratory leucocytes for transmission between co-feeding ticks (although there is some evidence that spirochaetes can be re-cultured from phagocytes following transport to the lymphatic system (Montgomery *et al.* 1993)). *Borrelia* spirochaetes likely rely on their periplasmic flagella that allow them to migrate autonomously through the tissues of the reservoir host (Charon *et al.* 2012). Co-feeding transmission of *Borrelia* spirochaetes may also benefit from saliva-assisted transmission (SAT) (Nuttall and Labuda, 2004), as this phenomenon is known to enhance co-feeding transmission of tick-borne viruses (Labuda *et al.* 1993*c*).

### Saliva-assisted transmission and co-feeding transmission

Ticks use their saliva to modulate the haemostatic, inflammatory and immune responses of the hosts and thereby optimize blood uptake (Brossard and Wikel, 2004). Tick saliva contains a wide variety of pharmacologically active agents that suppress both the innate and the acquired immune system of the vertebrate host (Nuttall, 1999; Nuttall and Labuda, 2004; Randolph, 2009). Tick saliva creates a zone of immunosuppression around the site of tick feeding that is beneficial to both the ticks and tick-borne pathogens. SAT thus refers to the phenomenon where saliva of the arthropod vector increases the transmission of vector-borne pathogens (Ribeiro, 1995). SAT and co-feeding transmissions are clearly connected; the pooled saliva of ticks feeding in close spatiotemporal proximity creates an environment that is propitious for co-feeding transmission. The two concepts are so closely linked that previous reviews considered co-feeding transmission as indirect evidence for SAT (Nuttall and Labuda, 2004).

The salivary gland extracts (SGE) from *I. ricinus* ticks suppresses both the innate and acquired immune response in their rodent hosts (Ribeiro and Spielman, 1986; Ribeiro, 1987; Ribeiro *et al.* 1990; Mejri *et al.* 2002; Pechová *et al.* 2002; Guo *et al.* 2009). This tick-induced immunosuppression is beneficial to the survival and fitness of *Borrelia* pathogens in the vertebrate host. For example, tick SGE from *I. ricinus* inhibited the ability of mouse macrophages to kill *B. afzelii* (Kuthejlová *et al.* 2001). Gern *et al.* (1993) provided some of the earliest evidence that the mode of inoculation (tick bite *vs* needle inoculation) influenced the dynamics of *Borrelia* infection and the immune response in laboratory mice. Later studies generated additional evidence that *Ixodes* tick SGE increase infectiousness and transmission of *Borrelia* pathogens. For example, *B. burgdorferi s. s*. uses its outer surface protein C (OspC) to bind the tick salivary gland protein Salp15, which allows the pathogen to evade the rodent immune response during the initial phase of the infection (Ramamoorthi *et al.* 2005). Co-inoculation of *Borrelia* pathogens with *Ixodes* tick SGE increased the spirochaete load in the tissues of laboratory rodents (Zeidner *et al.* 2002). Other studies have shown that spirochaete load in rodent tissues correlates with infectiousness (Wang *et al.* 2001*a*) and mouse-to-tick transmission (Raberg, 2012). Interestingly, the SAT effect was specific for the particular combination of *Ixodes* tick vector and *Borrelia* pathogen; *I. ricinus* SGE increased spirochaete load of a European but not an American *Borrelia* genospecies and vice versa for *I. scapularis* SGE (Zeidner *et al.* 2002). Another study found that co-inoculation of *B. afzelii* spirochaetes with *I. ricinus* SGE (via needle) resulted in efficient mouse-to-tick transmission to co-feeding nymphs (57%) whereas there was no mouse-to-tick transmission in the control mice that were inoculated with *B. afzelii* spirochaetes alone (Pechová *et al.* 2002). Thus tick-salivary gland products increase both tick-to-mouse and mouse-to-tick transmission rates of *Borrelia* pathogens.

Co-feeding transmission of *Borrelia* pathogens is different from TBEV because spirochaetes are capable of surviving in the skin for a substantial period of time following inoculation by tick bite. Previous work on *B. burgdorferi s. s*. showed that *Ixodes* ticks deposit spirochaetes into the skin where they multiply locally for about 1 week before disseminating to the rest of the body and establishing a systemic infection (Shih *et al.* 1992). More recent work found evidence for tick SGE effects on spirochaete population growth (Rudolf *et al.* 2003, 2010) and chemotactic behaviour (Shih *et al.* 2002), and both of these phenomena could facilitate co-feeding transmission. All three pathogenic *Borrelia* genospecies (*B. garinii, B. afzelii* and *B. burgdorferi s. s*.) grow faster *in vitro* in the presence of *I. ricinus* SGE (Rudolf *et al.* 2003, 2010). Again, the SAT effect is specific for the tick vector and SGE from non-competent vector ticks such as *Dermacentor reticulatus* did not enhance spirochaete population growth *in vitro* (Rudolf *et al.* 2003). With respect to chemotaxis, work on *B. burgdorferi s. s*. found that spirochaetes can migrate at substantial speeds (2 cm/day) through semi-solid media towards *Ixodes* tick SGE (Shih *et al.* 2002). The hallmark symptom of Lyme disease, erythema migrans, is further evidence that *Borrelia* pathogens migrate through the skin before disseminating and establishing a systemic infection. Another study found that *B. burgdorferi s. s*. spirochaetes respond to vertebrate host neuroendocrine stress hormones such as epinephrine and norepinephrine that are likely to be released at the tick feeding site (Scheckelhoff *et al.* 2007). Taken together, these studies suggest that the adaptive effects of SGE on spirochaete growth and chemotactic behaviour could easily be co-opted at the host-nymph-larva-pathogen interface to produce co-feeding transmission.

## ADAPTIVE SIGNIFICANCE OF CO-FEEDING TRANSMISSION

### Theoretical models of co-feeding transmission

The reproductive number of a parasite, *R*_0_, is a critical parameter in epidemiology. For directly transmitted infectious diseases, *R*_0_ is the number of secondary cases produced by a single infected individual when the host population is entirely susceptible. *R*_0_ therefore measures the capacity of a parasite to invade a population of susceptible hosts. For a tick-borne disease, the interpretation of *R*_0_ is complicated by the fact that there is a tick-to-host and a host-to-tick transmission step. However, in this case *R*_0_ represents the number of infected ticks produced by one infected tick in the previous generation. Recent theoretical work has used the next-generation matrix method to calculate *R*_0_ for tick-borne pathogens (Hartemink *et al.* 2008; Harrison *et al.* 2011; Harrison and Bennett, 2012). These theoretical analyses generally show that whereas co-feeding transmission is critical for TBEV, systemic transmission is sufficient for *Borrelia* pathogens to invade a population of susceptible hosts (Hartemink *et al.* 2008; Harrison *et al.* 2011; Harrison and Bennett, 2012). When ticks had a coincident, aggregated distribution, the value of *R*_0_ increased by 2·07 to 6·68% depending on the proportion of competent hosts (10–100%) from which the ticks derive their meal (Harrison and Bennett, 2012). This analysis suggests that a mutant genotype capable of both systemic and co-feeding transmission would be able to outcompete and eventually displace a resident genotype that uses systemic transmission alone. Thus co-feeding transmission may give *Borrelia* pathogens a competitive advantage in the context of mixed infections (see below).

Randolph *et al*. (1996) were the first to point out that the duration of infection is the main reason why TBEV (2 days) is critically dependent on co-feeding transmission whereas *Borrelia* pathogens (120 days) are not. Elasticity analysis of the next generation matrices of tick-borne pathogens have confirmed that *R*_0_ value of tick-borne pathogens is highly dependent on the duration of systemic infection (Hartemink *et al.* 2008). Changing the duration of systemic infection from 120 days to 2 days essentially switched the major contribution to *R*_0_ from systemic to co-feeding transmission (Randolph *et al.* 1996). It should be pointed out that all recent theories (Hartemink *et al.* 2008; Harrison *et al.* 2011; Harrison and Bennett, 2012) have used the same parameter estimates from the 1996 analysis by Randolph *et al*. All these theoretical studies therefore assume that the average duration of *Borrelia* infection is 120 days and that the host-to-tick transmission rate is 50% and constant over the age of the infection (Hartemink *et al.* 2008; Harrison *et al.* 2011; Harrison and Bennett, 2012). These parameter estimates were taken from early studies on competent rodent reservoir hosts that documented chronic infection and high rates of mouse-to-tick transmission (Donahue *et al.* 1987; Gern *et al.* 1994). However, other studies have shown that the mouse-to-tick transmission rate can decrease rapidly over time (Lindsay *et al.* 1997; Derdakova *et al.* 2004; Hanincova *et al.* 2008). For example, mouse-to-tick transmission of *B. burgdorferi s. s*. strain B348 declined from 80 to 0% over 42 days (Derdakova *et al.* 2004). Another study found that mouse-to-tick transmission declined from 75 to 26% in only 21 days (Lindsay *et al.* 1997). Incorporating this shorter duration of infectiousness would obviously increase the importance of co-feeding transmission to the fitness of *Borrelia* pathogens.

### Life history perspective of co-feeding transmission

From a life history theory perspective, the distinction between co-feeding and systemic transmission is similar to the trade-off between early and late reproduction that is common to all organisms (Stearns, 1992). On the one hand, systemic transmission is more efficient than co-feeding transmission suggesting that spirochaetes should maximize investment in systemic transmission to achieve the highest possible fitness. On the other hand, vulnerable reservoir hosts such as rodents have many sources of mortality (accidents, predators and disease) and dead rodents cannot transmit systemic infections. In addition, systemically infected individuals may disperse to new habitats that do not support larval ticks to complete the systemic infection cycle. Thus investment in co-feeding transmission may represent a bet-hedging strategy for the spirochaete because the future is uncertain and a systemic infection may not always bear fruit. As mentioned previously, numerous studies on *B. burgdorferi s. s*. have shown that the efficiency of mouse-to-larva transmission decreases with the age of the systemic infection in the reservoir host (Lindsay *et al.* 1997; Derdakova *et al.* 2004; Hanincova *et al.* 2008). Thus the fitness advantage of systemic transmission appears to decline with the age of the infection.

### Co-feeding transmission and the evasion of host immunity

Co-feeding transmission allows tick-borne pathogens to escape the host immune response that is directed at systemic infections. Immune evasion via co-feeding was first demonstrated in TBEV; the virus was still able to achieve co-feeding transmission on rodents that had developed virus-specific neutralizing antibodies in response to an earlier viraemic infection (Labuda *et al.* 1997). From an epidemiological perspective, hosts that had acquired resistance to systemic infection were still competent for co-feeding transmission.

The host immune system of vertebrate reservoir hosts likewise poses a major challenge for *B. burgdorferi s. l.* pathogens. Both the innate and the acquired arms of the vertebrate immune system can prevent the establishment of systematic spirochaete infections. The innate complement system is a collection of host serum proteins that assemble on the pathogen surface to form the so-called membrane attack complex, which is capable of puncturing the plasma membranes resulting in cell lysis and pathogen death. In the European Lyme disease system, host complement appears to play an important role in structuring associations between *Borrelia* pathogens and their vertebrate hosts (Kurtenbach *et al.* 1998*b*, 2002*a*). *Borrelia afzelii* and *B. burgdorferi s. s*. are tolerant of rodent but not bird complement (Kurtenbach *et al.* 1998*b*, 2002*a*) and are mostly found in rodent reservoir hosts (Humair *et al.* 1995; Humair and Gern, 1998; Kurtenbach *et al.* 1998*a*; Huegli *et al.* 2002; Hanincova *et al.* 2003*a*). Conversely, *B. garinii* and *B. valaisiana* are tolerant of bird but not rodent complement (Kurtenbach *et al.* 1998*b*, 2002*a*) and are mostly found in birds (Humair *et al.* 1998; Kurtenbach *et al.* 1998*a*,  2002*b*; Hanincova *et al.* 2003*b*; Taragel'ová *et al.* 2008). Vertebrate complement therefore plays an important role in restricting the range of reservoir hosts that are competent for systemic transmission.

Since its initial discovery, numerous authors have suggested that co-feeding transmission may allow *Borrelia* pathogens to derive some fitness gains from the otherwise incompetent hosts (Randolph *et al.* 1996; Gern *et al.* 1998) and there is some evidence to suggest that this is the case. For example, *B. garinii* and *B. valaisiana* achieved transmission between immature *Ixodes* ticks co-feeding on laboratory mice even though these *Borrelia* genospecies are generally killed by rodent complement (Sato and Nakao, 1997; Hu *et al.* 2003). A recent study on birds found that ticks co-feeding with other ticks were four times more likely to be infected with *B. afzelii* (Hasle, 2013). To date, the most convincing example comes from the northwest UK where co-feeding *I. ricinus* ticks maintain *Borrelia* pathogens in populations of sheep that are otherwise refractory to developing systemic spirochaete infections (Ogden *et al.* 1997).

Cervids are of particular interest with respect to co-feeding transmission because these animals are known to feed a large number of both immature and adult ticks (Jaenson and Talleklint, 1992; Matuschka *et al.* 1993). Recent work using host blood meal identification has confirmed the importance of deer as hosts for immature ticks in both North America and Europe. These studies found that 26·2–40·0% of all questing *Ixodes* nymphs obtained their blood meals from deer (and related artiodactyls) (Morán Cadenas *et al.* 2007; Scott *et al.* 2012). Earlier work on cervids suggested that these animals rarely transmitted *B. burgdorferi s. l.* to *Ixodes* ticks (Telford *et al.* 1988; Jaenson and Talleklint, 1992; Matuschka *et al.* 1993) but these studies did not consider the possibility of co-feeding transmission. A recent field study found that all stages of *I. ricinus* were highly clustered on roe deer suggesting that these animals could provide a platform for co-feeding transmission (Kiffner *et al.* 2011). An earlier field study on a variety of cervids found that 28·0% (14/50) of the animals had skin biopsies that tested positive for *B. burgdorferi s. l.* spirochaetes (Pichon *et al.* 2000). This study suggested that *Borrelia* spirochaetes can survive in cervid skin for a considerable period of time because the animals were shot in the winter when there is no tick questing activity (Pichon *et al.* 2000). A study on sika deer found that *I. persulcatus* ticks co-feeding on deerskin had a prevalence of *B. burgdorferi s. l.* that was five times higher than the background prevalence in questing nymphs (Kimura *et al.* 1995). The authors also showed that the spirochaetes in the sika deer-derived ticks were viable by culturing them in BSK medium (Kimura *et al.* 1995). This result was important because other *in vitro* studies have shown that *Borrelia* pathogens are generally killed by the ungulate complement (Kurtenbach *et al.* 1998*b*, 2002*a*). Host blood meal identification in questing ticks has found contradictory results with respect to whether deer can transmit viable spirochaete infections (Gray *et al.* 1999; Pichon *et al.* 2003, 2005; Morán Cadenas *et al.* 2007). An earlier study in Ireland found that all nymphs that had fed on deer were devoid of *Borrelia* spirochaetes (Gray *et al.* 1999). In contrast, a later study in Switzerland, found that that 18·4% (16/87) of all infections with *B. burgdorferi s. l.* occurred in nymphal ticks that had fed on artiodactyls (deer and chamois) (see Table 4 in Morán Cadenas *et al.* 2007). In summary, whereas earlier studies concluded that deer rarely transmitted *B. burgdorferi s. l.* to feeding ticks (Telford *et al.* 1988; Jaenson and Talleklint, 1992; Matuschka *et al.* 1993) the more recent work on host blood meal identification suggests that cervids can transmit viable spirochaete infections to *Ixodes* nymphs (Morán Cadenas *et al.* 2007). The host blood meal identification work currently suffers from low sensitivity (the blood meal is not identifiable for many questing ticks) and so the sample sizes are still relatively low. Future studies will hopefully establish with more certainty whether co-feeding transmission on cervids makes an important contribution to *Borrelia* fitness.

The acquired immune response can also prevent the establishment of systemic infections in otherwise competent reservoir hosts. Active and passive immunization of rodents with *Borrelia* pathogens induces an antibody response that prevents secondary infection by antigenically similar spirochaete strains (Johnson *et al.* 1986*a*, *b*; Piesman *et al.* 1997; Barthold 1999). In a natural population of *P. leucopus* mice, the anti-*Borrelia* antibody profile becomes increasingly hostile to new systemic infections over the course of the summer (Bunikis *et al.* 2004). Thus the likelihood that a tick-borne spirochaete can find a susceptible reservoir host becomes vanishingly small at the end of the summer. However, tick-borne *Borrelia* pathogens may still be able to derive some fitness gains from immune hosts if co-feeding transmission allows spirochaetes to escape the antibody response induced against a previous infection. A recent study on another tick-borne bacterial pathogen, the gram-negative, intracellular *A. phagocytophilum*, found that acquired immunity in *P. leucopus* reduced but did not eliminate co-feeding transmission (Levin and Fish, 2000). Surprisingly, to date, no one has tested whether acquired immunity reduces the efficiency of co-feeding transmission in *Borrelia* pathogens. The demonstration that acquired immunity blocks systemic but not co-feeding transmission would demonstrate the adaptive advantage of the latter in the context of acquired immunity in the vertebrate host.

### Advantage of co-feeding transmission in multiple infections

Co-feeding may be particularly important in the context of mixed infections where competition among strains will select for any additional transmission advantage. Previous studies have repeatedly shown that mixed infections of *Borrelia* strains are common in both the tick vector (Qiu *et al.* 1997, 2002; Wang *et al.* 1999; Pérez *et al.* 2011; MacQueen *et al.* 2012) and the rodent reservoir (Brisson and Dykhuizen, 2004; Swanson and Norris, 2008; Pérez *et al.* 2011; Andersson *et al.* 2013). A recent experimental infection study found that there was genetic variation in co-feeding transmission among nine strains of *B. afzelii* (Tonetti and Gern, 2011). Of the six strains that were capable of this mode of transmission, the efficacy of co-feeding transmission ranged between 3·8 and 66·2% (Tonetti and Gern, 2011). The *B. afzelii* strain that had the highest rate of co-feeding transmission (strain YU) had been discovered in a previous field study where it dominated the community of *B. afzelii* strains at the site with the higher level of coincident feeding between nymphal and larval ticks (Pérez *et al.* 2011). This field study thus suggested that co-feeding transmission can shape the community of *B. afzelii* strains, although there are alternative explanations (Pérez *et al.* 2011). For example, strains with high co-feeding transmission also have high tick-to-host and systemic (host-to-tick) transmission (Tonetti and Gern, 2011) suggesting that some *B. afzelii* strains are simply better at all the components of the spirochaete life cycle. The demonstration that there is genetic variation in co-feeding transmission among *Borrelia* strains is important because it shows that this trait can evolve by natural selection (Tonetti and Gern, 2011).

### Co-feeding facilitates co-occurrence of ecologically separated Borrelia species

Co-feeding transmission may facilitate encounters between *Borrelia* species that occupy different ecological niches in the community of vertebrate reservoir hosts. In Europe, as explained previously, the two most common *Borrelia* species, *B. afzelii* and *B. garinii*, are adapted to rodents and birds, respectively (Gern and Humair, 1998; Humair and Gern, 2000; Gern and Humair, 2002), and this host-pathogen specificity is mediated by vertebrate complement (Kurtenbach *et al.* 1998*b*, 2002*a*). Statistical analysis of the frequencies of single and double infections in wild ticks supports the hypothesis that *B. afzelii* and *B. garinii* occupy different ecological niches (Kurtenbach *et al.* 2001; Pichon *et al.* 2003; Herrmann *et al.* 2013). However, this ecological separation is not 100% complete and the two *Borrelia* species, by virtue of being common, encounter each other in the tick vector with appreciable frequency (Kurtenbach *et al.* 2001; Pichon *et al.* 2003; Herrmann *et al.* 2013). Co-feeding transmission is a plausible explanation for these co-infected nymphs (Kurtenbach *et al.* 2001; Pichon *et al.* 2003; Herrmann *et al.* 2013). For example, a larva may co-feed with a *B. garinii*-infected nymph on a *B. afzelii*-infected rodent reservoir host. In this example, the larva acquires *B. garinii* from the co-feeding nymph and *B. afzelii* from the rodent reservoir. The larval tick also ingests the host complement, which is active in the tick midgut (Papatheodorou and Brossard, 1987). The complement hypothesis of vertebrate host-*Borrelia* pathogen specificity predicts that the complement of the reservoir host (i.e. the rodent) would reduce the spirochaete load of the co-feeding-acquired *Borrelia* species (i.e. *B. garinii*) inside the larval tick. Interestingly, a recent study on the joint spirochete loads of co-infecting *Borrelia* species inside *I. ricinus* nymphs found evidence consistent with this complement hypothesis (Herrmann *et al.* 2013). In summary, co-feeding transmission explains the co-occurrence in nymphs of *Borrelia* species that occupy different niches in the community of vertebrate hosts. These occasional encounters in the tick vector can have important macro-evolutionary consequences for *Borrelia* pathogens. For example, genetic analysis of the *ospC* gene in *B. burgdorferi s. s., B. afzelii* and *B. garinii*, found numerous instances of horizontal transfer between these three *Borrelia* species (Baranton *et al.* 2001). Thus co-feeding transmission may facilitate genetic exchange between *Borrelia* pathogens that are otherwise genetically isolated.

### Concluding remarks

Future studies should investigate co-feeding transmission in the Lyme disease systems where it is likely to be important. The synchronized phenologies of immature *I. ricinus* ticks in Europe and the common occurrence of nymphal and larval ticks on the same host suggest that co-feeding transmission is more important in European than North American Lyme disease systems. Previous studies on *B. afzelii* and the ease of working with rodent models suggest that the *B. afzelii* pathogen–*I. ricinus* tick vector–is the most tractable system for studying the ecological significance of co-feeding transmission. Future studies should test whether co-feeding transmission allows *Borrelia* pathogens to escape the acquired immune response of their vertebrate hosts and whether this mode of transmission confers a fitness advantage in the context of mixed infections.
